# Supplementary material for: Partial Substitution of Urea with Biochar Induced Improvements in Soil Enzymes Activity, Ammonia-Nitrite Oxidizers, and Nitrogen Uptake in the Double-Cropping Rice System
Source: Microorganisms. 2023 Feb 19;11(2):527. doi: 10.3390/microorganisms11020527 (PMC9959172; doi:10.3390/microorganisms11020527)
Supplement: Supplementary file 1 [file microorganisms-11-00527-s001.zip › microorganisms-2187338-supplementary.pdf]

Supplementary file (S1)

Primer information

Target group	Primer name	Primer sequence	Fragment length (bp)	References
**AOB**	amoA-1F	GGGGTTTCTACTGGTGGT	491	
	amoA-2R	CCCCTCKGSAAAGCCTTCTTC		[1]
**AOA**	Arch-amoAF	STAATGGTCTGGCTTAGACG	635	
	Arch-amoAR	GCGGCCATCCATCTGTATGT		[2]
**NOB**	F	TACATGTGGTGGAACA	485	
	R	CGGTTCTGGTCRATCA		[3]

Note: AOB; ammonia oxidizing bacteria, AOA; ammonia oxidizing archaea, NOB; nitrite oxidizing bacteria

Supplementary file (S2)

Polymerase chain reaction (PCR) reaction system

**Component**	**Volume**
2×PCR Master Mix	12.5 µl
Forward primer (10 µM)	0.5 µl
Reverse Primer (10 µM)	0.5 µl
Template (mixed DNA)	1 µl
ddH_2O	25 µl

Setting of primer amplification conditions: 94°C for 5 min; 94°C for 30 s, 60°C for 30 s, 72°C for 30 s, 35 cycles; 72°C for 10 min. The products were subjected to agarose gel electrophoresis as follows:

Amplification curve: 94℃ for 30 s; 94℃ for 10 s, 60℃ for 12 s, 72℃ 30 s for 40 cycles, 72℃ single point detection signal.

Thymine-Adenine (TA) cloning reaction system

Component	Volume
The purified PCR product/or PCR1 ul 1 kb control	1 µl
PMD-19T Vector	1 µl
10×Enhancer	1 µl
Sterilized water	10 µl

Fluorescent quantitative polymerase chain reaction (FQ-PCR) system

Component	Volume	Final concentration
SYBR Premix Ex Taq（2×）	10 µl	1×
Forward Primer (10 µM)	0.8 µl	0.4 µM
Reverse Primer (10 µM)	0.8 µl	0.4 µM
Genomic DNA	1 µl	
ddH_2O	20 µl	

Fluorescence quantitative PCR amplification conditions setting:

Amplification curve: 94℃ for 30 s; 94℃ for 10 s, 60℃ for 12 s, 72℃ 30 s for 40 cycles, 72℃ single point detection signal.

Dissolution curve: 95℃ 0 s, 65℃ 15 s, 95℃ 0 s, continuous detection signal.

References:

[1] Rotthauwe JH, Witzel KP, Liesack W (1997) The ammonia monooxygenase structural gene *amoA* as a functional marker: molecular fine-scale analysis of natural ammonia-oxidizing populations. Appl Environ Microbiol 63:4704-4712

[2] Nicol GW, Leininger S, Schleper C, Prosser JI (2008) The influence of soil pH on the diversity, abundance and transcriptional activity of ammonia oxidizing archaea and bacteria. Environ Microbiol 10:2966–2978

[3] Pester M, Maixner F, Berry D, Rattei T, Koch H, Lücker S, Nowka B, Richter A, Spieck E, Lebedeva E, Loy A, Wagner M, Daims H (2014) NxrB encoding the beta subunit of nitrite oxidoreductase as functional and phylogenetic marker for nitrite-oxidizing Nitrospira. Environ Microbiol 16:3055–3071
